# From a Digital Bottle: A Message to Ourselves in 2039

**DOI:** 10.2196/16274

**Published:** 2019-11-01

**Authors:** Alejandro R Jadad, Tamen M Jadad Garcia

**Affiliations:** 1 Dalla Lana School of Public Health University of Toronto Toronto, ON Canada; 2 Beati Inc Toronto, ON Canada

**Keywords:** wisdom, pandemic, concepts, future, extinction, self-sabotage, precariat, stupidity, noosphere, capitalism

## Abstract

We are fully aware that we could have wasted our time writing this message, as nobody might read it. Even those who read it might ignore it, and those who read and care about it might be unable to do anything. It may simply be too late. Nevertheless, this message describes the hopes we had back in 1999, imagining how the incredible digital tools whose birth we were witnessing, could change the world for the better. In 2019, when we wrote these words, we were saddened to realize that most of what we had imagined and proposed in the past 20 years could have been written the day before, without losing an iota of relevance. Whoever or whatever you might be, dear reader—a human, a sentient machine, or a hybrid—we would like you to understand that, rather than an attempt to predict the future, which probably continues to be an impossible endeavor, this message was meant to act as an invitation, regardless of when or where it is found, to engage in a conversation that has already transcended time and space, even if the issues it contains have become irrelevant.

To whom might read this, human or sentient machine:

We are fully aware that we could have wasted our time writing this message, as nobody might read it. Even those who read it might ignore it, and those who read and care about it might be unable to do anything. It may simply be too late.

We would like to share with you the hopes we had back in 1999, imagining how the incredible digital tools whose birth we were witnessing, could change the world for the better. As a Babyboomer father, who, as a physician, had started communicating with patients via email in 1990 and a Millennial daughter who saw ways to improve healthcare since her infancy [1], we have spent the last two decades dreaming about and proposing new possibilities that would enable everyone to enjoy a healthy life.

It saddens us to realize that most of what we had imagined and proposed in the past two decades could have been written yesterday, without losing an iota of relevance today.

Twenty years ago, we imagined new forms of partnerships between healthcare professionals and the public [2-4], enlightened by trustworthy information, available anywhere, in the right format, in the right amount, with the right balance [5-8]. We called for a major shift “from our ethic of competition and narrow self interest, focused on gadgets—to one of generosity and collaboration, centred on people.” [9]

Instead, we are now facing an avalanche of “fake” or misleading knowledge that is capable of reaching the masses more effectively than verified facts, particularly through social media [10]. This has opened the door to new spaces for hate speech and manipulation of public opinion, undermining trust and hindering efforts to control diseases that should have been eradicated already, or that could otherwise be managed easily [10-12].

In the past two decades, we dreamed about ways in which we could use every new technological development to create tailored services, available to all people, that could transcend our traditional geographic, cultural, religious, institutional, and political boundaries [13-15]. We believed that it was possible to join forces across the world to imagine and reinvent how we care for each other, through the enthusiastic and proficient use of information and communication technologies [16,17].

Instead, in 2019, we face a landscape littered by variations in the same disconnected examples that have been used for two decades to illustrate how digital tools could contribute to the prevention, diagnosis, or treatment of diseases. Sadly, the promises of information and communication technologies to transform healthcare services remain unfulfilled as a result of poorly designed interfaces, persistently increasing workload for providers, unresolved privacy concerns, lack of sensible models for the reimbursement of new services, chronically outdated communication skills, and obsolete rules of etiquette to guide interactions at all levels [18,19].

We also imagined new possibilities to unlearn, to “un-see” and “un-believe” in the barriers that prevent us from having a full life, from before our first breath to the last one [20]. We dreamed that we could look at death as the main source of insights that could inspire us to live fully, without fear, regrets or distress, through all stages of our lives [21-23].

Instead of broadening our understanding of the mental and social aspects of our lives, we kept steering our technological innovations to go deeper and deeper into the body. Since the turn of the 20th century, informatics has enabled an explosion in the biological understanding of what ails us, bringing back the dreams of yore about our capacity to personalize medical responses to physical challenges. Such insights, nevertheless, have also emboldened the medical industrial complex to boost its efforts to create sophisticated weapons to feed the war against diseases. The infatuation around “fixing” physical problems is now manifesting through an apparently inexhaustible menu of ever-reductionist “omics” [24,25], which are taking the chemical-mechanical view of humanity to new depths [26] while feeding a frantic new era of hypermedicalization of life. Instead of being a source of humility and insights, death has now become something for which there might be a “cure” [27].

During the past two decades, we used online platforms to nurture a global conversation about the meaning of health [28,29]. This led to a new conceptualization that views it as the ability, which anyone could develop, to adapt and manage the inevitable challenges we face through life as individuals or communities [30]. We invited the world to unleash a pandemic of health through the enlightened and generous use of digital technologies [31,32]. We viewed the internet as a treasure trove of opportunities to celebrate and boost the “high-touch” with the “high-tech” [16].

Rather than using them to usher in truly planet-wide, open, equitable, affordable services that are culturally sensitive and tailored to our unique needs and those of other living beings, sophisticated digital platforms are accelerating the privatization and segmentation of a “sickcare” system in practically every region of the world while transforming it into a branch of the financial system [33]. The same phenomenon of “financialization,” driven by global digital platforms and algorithms, has engulfed all other aspects of human life, compounding the threats to health at all levels, from the individual to the planetary level. Digital technologies are at the core of the engines that have made 2019 a year full of nefarious records in terms of global warming, major weather events, deforestation, and extinction of animal and plant species [34-37]. As a result, environmental factors have become the main cause of one quarter of all deaths in the world [38].

We hoped, above all, that digital technologies would enable us to prevail, transforming us into “humanodes” in a global superorganism, feeding a Noosphere—a planetary thinking network of reasoning minds—in which we all could divert our seemingly unavoidable course toward a world without humans to one in which we would thrive [39].

We were naive…

The opportunities we have had over the last 20 years appear to have been “insurmountable.”

Instead of realizing the dream of a humanity that could be connected deeply and meaningfully into an enlightened whole, technology over the past 20 years has been used, relentlessly, to dismantle communities and societies around the world, segmenting them all the way to the individual level [40]. This extreme level of “precision capitalism,” which is fueled by social media, is leading to unprecedented levels of isolation [41], making loneliness a growing source of preventable deaths [42] and low levels of well-being [43].

Our growing disconnection from each other is also facilitating the emergence of new economic models ruled by a progressively smaller group of unaccountable masters who have unleashed a growing network of all-powerful algorithms. Such models consider the vast majority of humans either as consumers of the goods or services they peddle or as biological automatons that are relevant only because they are capable of performing tasks that machines are still unable to complete or because they are less expensive [44].

The relegation of humans to the margins of the economy is breeding fresh threats to health, which multiply and reinforce each other as technology takes over every aspect of human life. A case in point is the impact that automation and artificial intelligence are having on existing occupations and on the ways in which humans make a living. As machines and artificial intelligence gain dominance, a new class of unfortunate humans is emerging, under the guise of freedom [45]. Known collectively as “the precariat,” these are people who are self-employed or freelancers, doing mostly “gig jobs” without long-term or permanent contracts [46]. Often, they are overqualified individuals who work under short-term or zero-hour contracts, sometimes for a few minutes at a time, without benefits provided by employers [47]. Even physicians are now at risk of joining this class [48].

With the pervasive use of digital technology driving people into precariousness, marketeers, engineers, and scientists at the service of the new unchecked overlords have also devised new tricks to extract the remaining disposable income available to the helpless masses. In 2019, people’s attention has become the main target [49]. As a result, digital devices—or, more precisely, the experiences they purvey—have been manipulated to become increasingly addictive, thanks to constant monitoring and experimentation on consumers through the platforms they use and the data they produce [50]. This has generated new problems, such as those associated with gaming, gambling, or over-consumption of social media, and in the invigoration of old ones, manifested through shopaholism, workaholism, eating disorders, online pornography, or the rapid proliferation of electronic cigarettes [51].

In 2019, the new threats created by the massive digitalization of the planet are finding all those charged with safeguarding or improving human health wanting. Front line healthcare providers, in particular, are ill equipped to face the vastly complex array of new challenges. In the past 20 years, instead of becoming examples of how to lead healthy lives, physicians and nurses are facing high levels of depression, stress, anxiety, and burnout [52-55]. The prevalence of addiction is likely similar or higher among physicians when compared with the general public [56]. They also commit suicide more often, with women at an even greater risk [57]. In fact, suicide is the only cause of death that has a higher prevalence among physicians than the general population. Male physicians are 40% more likely than members of the general public to kill themselves, while the risk is more than double for female physicians [58]. The situation among nurses is likely similar [59,60].

As we kill ourselves in ever more creative ways, the ability of machines to control those who will remain alive is increasing. As the so-called “The Internet of Things” materializes, a practically unimaginable number of interconnected devices, compounded by our cavalier ways to share our information with corporations, governments and financial institutions is curtailing our freedom. Insidiously, we are becoming prisoners in a digital panopticon run by a surveillance capitalist apparatus [61]. Given that it is estimated that the number of connected computing devices will exceed one trillion in the next 20 years [62], we are facing a world in which instead of humans controlling the Internet of Things, we could become one of the many things controlled by the internet [63].

We can no longer afford to be oblivious to what is obvious.

The space for humanity to flourish in the middle of a spectrum spanning from apocalyptic and transhumanist extremes has narrowed significantly in the past 20 years [64]. We might be facing the worst possible scenario: a self-inflicted apocalyptic posthuman era, brought about by our seemingly unstoppable propensity to self-sabotage.

Instead of using our digital offspring to nurture the Noosphere, we have created the “Atesphere”—after Ate, the goddess of delusion, blind folly, and ruin [65]—an interconnected planetary layer of human stupidity, fed by our self-harming tendencies. Within it, we are hastening our complete or near-complete extinction.

To counterbalance the risk that such a pessimistic stance could lead to a self-fulfilling prophecy, we would like to put forward a counter argument [66]: It is also possible for humans and machines to create a “Sophosphere,” a planetary network of interconnected people, machines, and all other living things striving to negate self-sabotage through wisdom.

We believe that wisdom could be the best antidote to self-sabotage because, based on a conceptualization proposed in 1999 [67], we view it as “the ability to know what it is to live well, and do everything possible to achieve this, given the circumstances, while enabling others to do the same, as a means to overcome the destructive mental processes and behaviours that inhibit our own ability to experience a full life, in our own way, together.” This approach, which we feel adds balance to the Atesphere, makes it possible for machines to be wise too and for machines and humans to collaborate and create the conditions needed to live well in harmony with other living things.

In a consistently mirror-like fashion, the Sophosphere might occur, as a Big Bang, in response to the devastating effects of the Atesphere at its peak. Just as it happens with individuals who undergo a near-death experience, it has been suggested that our species could go through a similar process, snapping back from the brink of death, en masse, to embrace radically different priorities and values and be ready to live fully [68].

Another option, also counterintuitive, involves doing nothing, on the basis of the belief that our frantic efforts to make change happen might be part of the problem, rather than part of the solution. Instead, this approach calls for restraint and patience, as it regards massive social change as a nonlinear phenomenon that could happen suddenly, in response to the accumulation of little adjustments made by large enough numbers of individuals or small groups to their own lives [69].

In addition, the Sophosphere could emerge more deliberately, through the collective efforts of innovators from different sectors, joining forces in and from different regions of the world to incubate new ways to coexist harmoniously, which could then spread, through social contagion, until they become pandemic [32].

As noted a few years ago, the most sensible option might be hardest to accept, as it requires recognizing that humanity is terminally ill as the result of self-inflicted wounds, and that the wisest course of action is to engage in palliative care measures at a species level [39].

Having reached this point, dear reader, whoever or whatever you might be, we would like you to understand that, rather than an attempt to predict the future, which is impossible, this message that was released into the cyberwaves in 2019 should be viewed as an invitation, regardless of when or where it is found, to engage in a conversation that has already transcended time and space, even if the issues it contains have become irrelevant.

We will only know what will happen when it happens.

## Appendix

Multimedia Appendix 1
