# Supplementary figures and images for: From a Digital Bottle: A Message to Ourselves in 2039
Source: J Med Internet Res. 2019 Nov 1;21(11):e16274. doi: 10.2196/16274 (PMC6858618; doi:10.2196/16274)

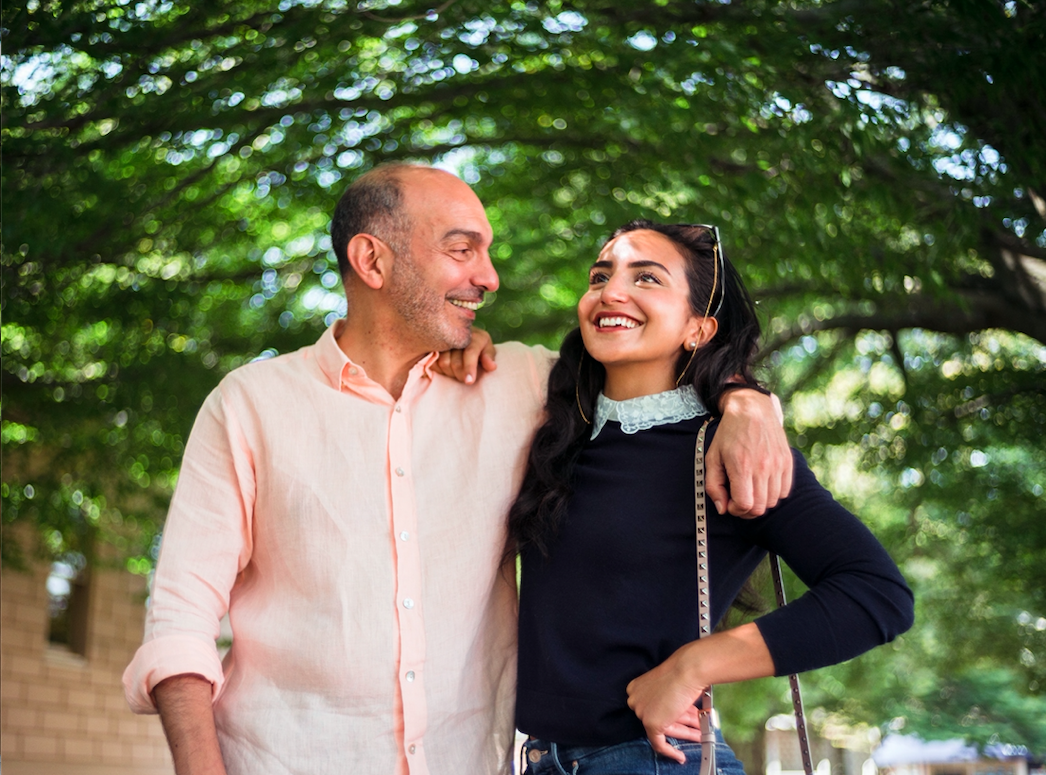

Supplement: Multimedia Appendix 1 [file jmir_v21i11e16274_app1.png]
